# Plasmonic Gold Nanoisland Film for Bacterial Theranostics

**DOI:** 10.3390/nano11113139

**Published:** 2021-11-21

**Authors:** Shih-Hua Tan, Sibidou Yougbaré, Hsuan-Ya Tao, Che-Chang Chang, Tsung-Rong Kuo

**Affiliations:** 1 Graduate Institute of Nanomedicine and Medical Engineering, College of Biomedical Engineering, Taipei Medical University, Taipei 11031, Taiwan; b812105028@tmu.edu.tw; 2 Institut de Recherche en Sciences de la Santé (IRSS-DRCO)/Nanoro, 03 B.P 7192, Ouagadougou 03, Burkina Faso; d845107003@tmu.edu.tw; 3 School of Biomedical Engineering, College of Biomedical Engineering, Taipei Medical University, Taipei 11031, Taiwan; b812107008@tmu.edu.tw; 4 The Ph.D. Program for Translational Medicine, College of Medical Science and Technology, Taipei Medical University, Taipei 11031, Taiwan; 5 International Ph.D. Program for Translational Science, College of Medical Science and Technology, Taipei Medical University, Taipei 11031, Taiwan; 6 International Ph.D. Program in Biomedical Engineering, College of Biomedical Engineering, Taipei Medical University, Taipei 11031, Taiwan

**Keywords:** gold nanoisland film, surface-enhanced Raman scattering, detection, photothermal therapy, theranostics

## Abstract

Plasmonic nanomaterials have been intensively explored for applications in biomedical detection and therapy for human sustainability. Herein, plasmonic gold nanoisland (NI) film (AuNIF) was fabricated onto a glass substrate by a facile seed-mediated growth approach. The structure of the tortuous gold NIs of the AuNIF was demonstrated by scanning electron microscopy and energy-dispersive X-ray spectroscopy. Based on the ultraviolet-visible spectrum, the AuNIF revealed plasmonic absorption with maximum intensity at 624 nm. With the change to the surface topography created by the NIs, the capture efficiency of *Escherichia coli* (*E. coli*) by the AuNIF was significantly increased compared to that of the glass substrate. The AuNIF was applied as a surface-enhanced Raman scattering (SERS) substrate to enhance the Raman signal of *E. coli*. Moreover, the plasmonic AuNIF exhibited a superior photothermal effect under irradiation with simulated AM1.5 sunlight. For photothermal therapy, the AuNIF also displayed outstanding efficiency in the photothermal killing of *E. coli*. Using a combination of SERS detection and photothermal therapy, the AuNIF could be a promising platform for bacterial theranostics.

## 1. Introduction

Plasmonic nanomaterials have been extensively utilized for diagnostic applications because of their unique properties, such as a large surface area, easy surface modification, and distinct surface plasmon resonance [1,2,3,4,5,6,7,8,9,10]. Among various plasmonic nanomaterials, gold-based nanomaterials with tunable size and shape have been extensively applied for biomedical detection based on their optical and structural characteristics [11,12,13,14,15,16,17]. For example, plasmonic gold nanoparticle (NP; AuNP) multilayers with a superior photothermal effect were fabricated as a sample substrate to assist the desorption and ionization of a bone biomarker, hydroxyproline, for its detection by mass spectrometry [18]. Peptide-nucleic-acid-conjugated gold nanorods were designed for the sequence-specific detection of circulating tumor DNA point mutations according to changes in their surface plasmon resonance absorbances [19]. A reliable naked-eye gold-nanoshell-enhanced immunoblotting approach was developed to detect the *Mycobacterium tuberculosis* antigen 85B due to the formation of gold nanoshells on the surface of AuNPs, which resulted in the production of a purple spot [20]. Gold nanostars deposited on carbon electrodes showed great enhancements in various factors such as the electroactive surface area, the geometric surface area, charge-transfer resistance, and double-layer capacitance for the electrochemical detection of arsenic, mercury, and lead [21]. The high-performance, surface-enhanced, Raman scattering (SERS) substrate of plasmonic AuNPs was explored to analyze the serum of people with and without cervical cancer [22]. Advancements made regarding plasmonic gold nanomaterials have revealed a promising potential for the biomedical detection of biomolecules, diseases, and heavy metals.

Plasmonic nanomaterials also demonstrated biomedical applications in phototherapy [23,24,25,26,27,28]. Based on strong photothermal effects, plasmonic gold nanomaterials have been intensively investigated for cancer therapy, wound healing, and antibacterial applications [29,30,31,32,33]. For example, a plasmonic gold nanocage-based anticancer nanoplatform that was photothermally responsive in the near-infrared (NIR) region was employed to treat MCF-7 tumors [34]. Nanomaterials of the lipid bilayer coated with reduced graphene oxide and gold nanostars exhibited outstanding anticancer efficacy against pancreatic cancer tumors in mice due to their synergistic photothermal and gene therapies [35]. Gold nanorods conjugated with polymers that had bacterial affinities were used to efficiently kill bacteria, resulting in the acceleration of wound healing in diabetic rats [36]. With similar surface plasma bands at ~808 nm, gold nanobipyramids with a (111) plane exhibited a higher photothermal efficiency compared to that of gold nanorods with a (200) plane for the photothermal killing of *Escherichia coli* [37]. Many efforts have been made to confirm the therapeutic applications of plasmonic gold-based nanomaterials with strong photothermal effects. However, developing multifunctional gold-based nanomaterials for bacterial theranostics is an area still urgently needing exploration for treating bacterial infections.

Recently, plasmonic gold nanoisland (NI) film (AuNIF) was fabricated to improve biomedical detection based on unique plasmon-enhanced light–matter interactions, including fluorescence enhancement, a photoacoustic effect, and SERS enhancement [38,39,40,41,42]. Furthermore, by taking advantage of its durable stability and extensive uniformity, the AuNIF was demonstrated to meet the requirements for practical biomedical detection. For instance, for the diagnosis and tracking of myocardial infarction, the AuNIF was applied to the ultra-sensitive analysis of the serum biomarker cardiac troponin I, with a 130-fold enhancement by NIR fluorescence [43]. A multiplex serology assay that used the AuNIF was performed to detect *Toxoplasma gondii*, rubella immunoglobulin G (IgG), and cytomegaloviruses in serum, whole-blood, and saliva samples by plasmon-enhanced fluorescence [44]. The AuNIF immobilized with Zika virus (ZIKV) and dengue virus (DENV) antigens was used to detect IgG and IgA antibodies and IgG avidity to diagnose Zika virus and dengue virus infections [45]. Although the AuNIF was validated to have diverse capabilities for disease detection, there are still only a few investigations of photothermal therapy based on the AuNIF.

In this work, a facile seed-mediated growth approach was used to deposit the AuNIF onto a glass substrate of a Polysine slide. The structural and optical characteristics of the AuNIF were analyzed by scanning electron microscopy (SEM), energy-dispersive X-ray (EDX) spectroscopy, and ultraviolet-visible (UV-Vis) spectroscopy. The AuNIF was applied as a SERS substrate to enhance Raman signals of *E. coli*. Photothermal effects of the AuNIF were examined by irradiation with simulated AM1.5 sunlight. Photothermal therapy of *E. coli* by the AuNIF was quantitatively measured using fluorescence microscopy.

## 2. Materials and Methods

### 2.1. Materials

Glass substrates of Polysine slides (2.5 cm × 7.5 cm × 0.1 cm), dimethylformamide (DMF, 99.5%) and SYTOX green were purchased from Thermo Fisher Scientific (Waltham, MA, USA). Succinic anhydride (99%) was purchased from AK Scientific (Union City, CA, USA). *N*,*N*-Diisopropylethylamine (DIPEA, 98%) and sodium borohydride (NaBH_4_, 98%) were purchased from Acros (Morris, NJ, USA). Hydrogen tetrachloroaurate (III) trihydrate (HAuCl_4_·3H_2_O) and hydroxylamine hydrochloride (H_3_NO·HCl) were purchased from Alfa Aesar (Haverhill, MA, USA). An ammonium hydroxide solution (NH_4_OH, 30%) was purchased from Honeywell Fluka^TM^ (Loughborough, UK). Lysogeny broth (LB) (Miller), kanamycin, yeast extract (pH 7.0 ± 0.5), and peptone were purchased from Bioshop Canada (Burlington, ON, Canada). D(-)-Mannitol (C_6_H_14_O_6_) was purchased from PanReac AppliChem ITW Reagents (Barcelona, Spain). Paraformaldehyde (PFA) was purchased from Sigma-Aldrich (St. Louis, MO, USA).

### 2.2. Preparation of the AuNIF

A Polysine slide was utilized as a glass substrate to prepare the AuNIF. Before preparation of the AuNIF, the Polysine slide was sequentially cleaned with acetone, methanol, and deionized (DI) water under ultrasonication. After the washing processes, the Polysine slide was modified with carboxylic groups by immersing it in a solution containing 0.45 g of succinic anhydride, 25 mL of DMF, and 0.606 mL of DIPEA in an orbital shaker at 25 rpm and 25 °C for 18 h in the dark. Afterward, the glass substrate was rinsed with ethanol and DI water. To deposit gold ions (Au^3+^), the carboxylic-modified glass substrate was first immersed in a solution containing HAuCl_4_ (3 mM) and NH_4_OH (0.6%) under shaking at 40 rpm and 10 °C for 20 min. After 20 min, the glass substrate was rinsed twice with DI water and then moved to a beaker containing 50 mL of DI water at 10 °C. The gold ions adsorbed onto the glass substrate were further reduced to gold seeds (Au^0^) by adding 50 mL of NaBH_4_ (4 mM) under shaking at 40 rpm and 10 °C for 5 min. After reduction, the glass substrate with gold seeds was washed with DI water. For growth of the AuNIF, the glass substrate with gold seeds was mixed with HAuCl_4_ (500 μM) and NH_2_OH (500 μM) under shaking at 40 rpm and 30 °C for 5 min and then left undisturbed for another 10 min. The glass substrate with the AuNIF was rinsed with DI water and further spin-dried. The glass substrate with the AuNIF was stored at room temperature with protection from light for the following experiments.

### 2.3. Culture of Bacteria onto the Surface of the AuNIF

*Escherichia coli* was activated and cultured in LB at 37 °C on a shaker at 170 rpm. Afterward, the glass substrate with the AuNIF was immersed into 2 mL of the *E. coli* suspension with an OD600 value of 0.1 at 37 °C for 30 min. After 30 min, the AuNIF cultured with *E. coli* was rinsed with DI water to remove the culture medium. To examine the morphology by SEM, the AuNIF with *E. coli* was fixed with 4% PFA.

### 2.4. Bacterial Growth under Different Temperatures

*Escherichia coli* suspensions with an OD600 value of 0.1 were cultured at various temperatures, including 37, 45, 50, 55, and 60 °C while shaking at 170 rpm. During culture, the OD600 values of the *E. coli* suspensions were measured every 30 min to evaluate the effect of temperature on the bacterial growth rate.

### 2.5. Bacterial Inhibition by the AuNIF under Light Irradiation

To investigate bacterial inhibition under light irradiation, the glass substrate with the AuNIF was first cultured with an *E. coli* suspension with an OD600 value of 0.1 in culture dishes at 37 °C for 2 h. Afterward, the AuNIF with *E. coli* was irradiated by simulated AM1.5 sunlight. After light irradiation, the death of *E. coli* was evaluated by fluorescence imaging. For fluorescence imaging, the AuNIF with *E. coli* was incubated with SYTOX green nucleic acid stain (5 μM) in a culture dish for 30 min. Next, Hoechst 33342 (15 μg/mL) was added to the culture dish and incubated further for 30 min. All staining procedures were executed in the dark. After the staining processes, the AuNIF with *E. coli* was immersed in phosphate-buffered saline (PBS) buffer for 10 min to remove any remaining SYTOX green and Hoechst 33342 dyes. The washing process was repeated three times. *Escherichia coli* on the AuNIF was observed under a fluorescence microscope (Leica DMi8) (Wetzlar, Germany), where dead *E. coli* was stained with SYTOX green (producing a green pseudocolor), and total *E. coli* was stained with Hoechst 33342 (producing a blue pseudocolor).

## 3. Results

### 3.1. Characterizations of the AuNIF

**Figure 1:**
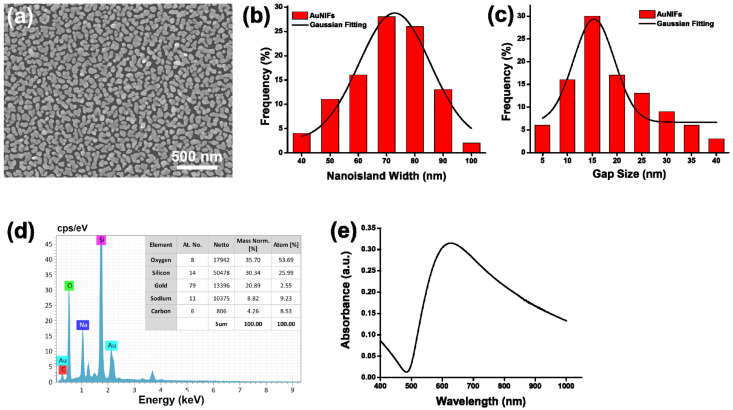
(**a**) SEM image of the gold nanoisland film (AuNIF), (**b**) the histogram of nanoisland width of AuNIF and its Gaussian-fitting curve, (**c**) the histogram of nanoisland gap of AuNIF and its Gaussian-fitting curve, (**d**) EDX spectrum of the AuNIF and elemental composition (inset table), and (**e**) UV-Vis spectrum of the AuNIF.

The AuNIF was structurally and optically characterized by SEM, EDX, and UV-Vis spectroscopy. As shown in the SEM image of Figure 1a, the AuNIF revealed convoluted NIs over the entire substrate. To evaluate the average width of the NIs of the AuNIF, a histogram and a related Gaussian-fitting curve of the width distribution of 100 NIs in the SEM image were calculated as shown in Figure 1b. The simulation result of the Gaussian fitting curve for the NI width indicated that the average width of the NIs was ca. 72.9 ± 1.37 nm. Furthermore, based on 100 gaps in the SEM image, the average gap between the NIs was calculated to be 15.4 ± 0.76 nm, according to the histogram and Gaussian-fitting curve shown in Figure 1c. Moreover, in Figure 1d, the EDX analysis of the AuNIF on the glass substrate revealed that the weight percentage of gold was 20.89%. The EDX spectrum of the AuNIF demonstrated the successful deposition of gold NIs on the glass substrate. In Figure 1e, the UV-Vis spectrum of the AuNIF exhibited a broad absorption with maximum intensity at 624 nm due to surface plasma absorption. Overall, results of the structural and optical characterization demonstrated that the plasmonic AuNIF could be a potential SERS substrate for bacterial detection.

### 3.2. The AuNIF as a SERS Substrate for Bacterial Detection

**Figure 2:**
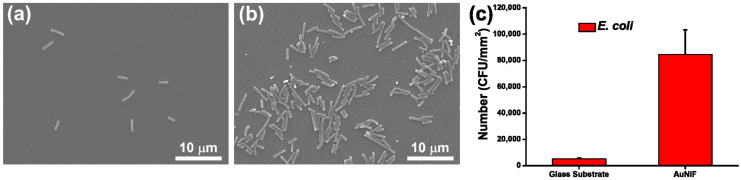
SEM images of *Escherichia coli* on surfaces of (**a**) the glass substrate and (**b**) the gold nanoisland film (AuNIF). (**c**) Statistical calculation of *E. coli* on the surfaces of the glass substrate and the AuNIF.

To examine the capability of the AuNIF as a SERS substrate for bacterial detection, *E. coli* was separately cultured on the surfaces of the glass substrate and the AuNIF. As shown in an SEM image of Figure 2a, a few *E. coli* were randomly attached to the glass substrate. Compared to the glass substrate, the number of attached *E. coli* was significantly higher on the AuNIF surface, as shown in Figure 2b. The increased number of attached *E. coli* on the AuNIF can be attributed to its surface topography of NIs [46]. Moreover, the length of the *E. coli* on the AuNIF was greater because the attached *E. coli* were stretched due to the strong interactions between the *E. coli* and the AuNIF surface [47]. Furthermore, in a quantitative evaluation of the bacterial capture efficiency, numbers of *E. coli* on the glass substrate and the AuNIF were separately calculated, as shown in Figure 2c. Average numbers of *E. coli* on the glass substrate and AuNIF were, respectively, calculated to be 5066 and 84,500 colony-forming units (CFU)/mm^2^. The bacterial capture efficiency of the AuNIF was 16.7-fold higher than that of the glass substrate. Results of the bacterial capture efficiency indicate that the AuNIF could be a substrate with a high capture efficiency of bacteria for further application in bacterial SERS detection.

**Figure 3:**
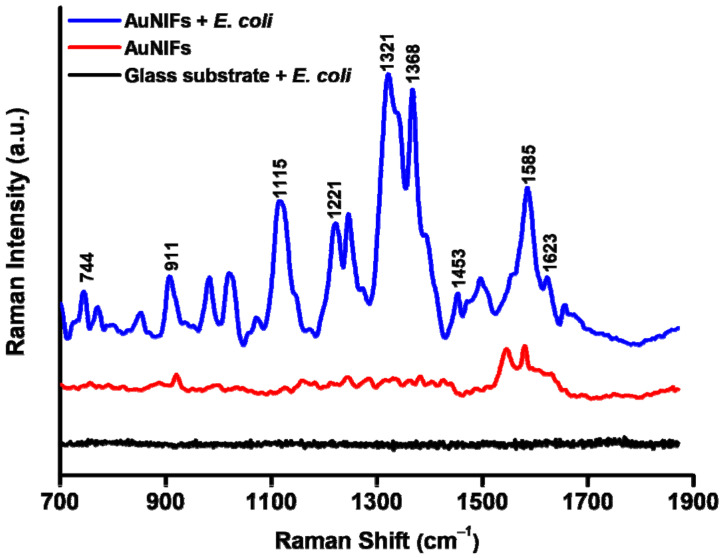
Raman spectra of the glass substrate cultured with *Escherichia coli* (black line), the gold nanoisland film (AuNIF) without *E. coli* (red line), and the AuNIF cultured with *E. coli* (blue line).

To detect bacteria, the glass substrate and AuNIF with cultured *E. coli* were measured by Raman spectroscopy. As shown in Figure 3, the glass substrate with cultured *E. coli* exhibited no obvious Raman signal of *E. coli*. For the AuNIF without *E. coli*, there were two Raman signals at 1543 and 1579 cm^−1^ due to the characteristic peak of carboxyl groups on the surface of the AuNIF. After culturing *E. coli* on the AuNIF, the characteristic Raman signals of *E. coli* were observed at 744, 911, 1115, 1221, 1321, 1368, 1453, 1585, and 1623 cm^−1^ (Table 1) [48,49,50]. Results of Raman measurements indicate that the plasmonic AuNIF can be applied as a promising SERS substrate that enhances *E. coli* Raman signals.

### 3.3. The AuNIF for Photothermal Therapy of Bacteria

**Figure 4:**
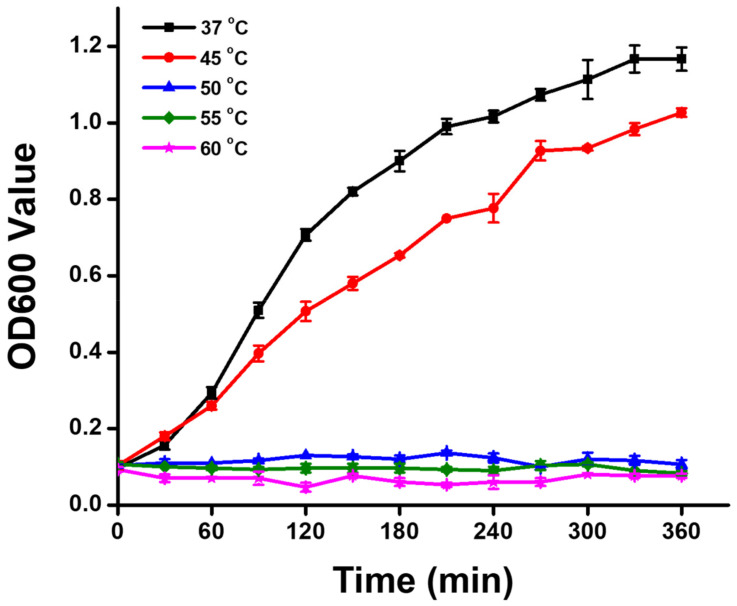
Growth curves of *Escherichia coli* at different culture temperatures. OD600 values of *E. coli* were measured every 30 min.

To investigate photothermal therapy, *E. coli* was cultured at various temperatures of 37, 45, 50, 55, and 60 °C to examine the influence of temperature on its growth. As shown in the growth curves of Figure 4, for a normal culture temperature at 37 °C, *E. coli* revealed exponential growth, and after culture for 360 min, the OD600 value of *E. coli* reached 1.17. At a culture temperature at 45 °C, slight growth inhibition of *E. coli* was noted, and after culture for 360 min, the OD600 value of *E. coli* was 1.03. Most importantly, at culture temperatures of 50, 55, and 60 °C, the growth curves of *E. coli* indicated no significant growth. After culturing for 360 min, the OD600 values of *E. coli* cultured at 50, 55, and 60 °C were 0.10, 0.08, and 0.08, respectively. Results of *E. coli* cultured at different temperatures showed that the *E. coli* used in this work could not survive at temperatures above 50 °C.

**Figure 5:**
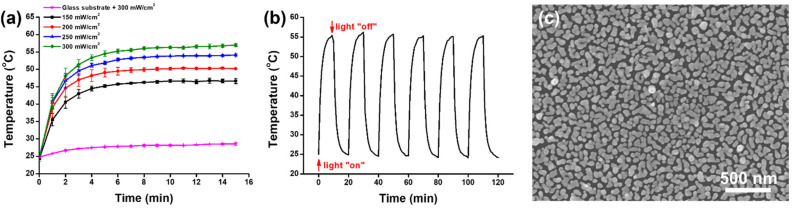
(**a**) Photothermal effects of the glass substrate and the gold nanoisland film (AuNIF) under simulated AM1.5 sunlight irradiation. (**b**) Photothermal performance of the AuNIF under on/off cycles of simulated AM1.5 sunlight irradiation at a power density of 300 mW/cm^2^. (**c**) SEM image of the AuNIF after the on/off cycles of the simulated AM1.5 sunlight irradiation at power density of 300 mW/cm^2^.

To evaluate the photothermal effect, the AuNIF was irradiated by simulated AM1.5 sunlight at different power densities of 150, 200, 250, and 300 mW/cm^2^. For the control experiment, the glass substrate was irradiated with simulated AM1.5 sunlight at 300 mW/cm^2^. The temperatures of the AuNIF and the glass substrate were measured by a thermal camera (FLIR TG267) (Teledyne FLIR, Wilsonville, CA, USA). As shown in Figure 5a, the temperature of the glass substrate revealed no significant increase under light irradiation. However, with light irradiation at power densities of 150, 200, 250, and 300 mW/cm^2^ for 15 min, temperatures of the AuNIF were measured to be 46.6, 50.2, 54.1, and 60.0 °C, respectively. Most importantly, with light irradiation at a power density of 300 mW/cm^2^ for 10 min, the temperature of the AuNIF increased to 56.3 °C to reach the requirement for the photothermal killing of bacteria. Moreover, to demonstrate the reversibility of the photothermal effect, the AuNIF was first irradiated by light at a power density of 300 mW/cm^2^ for 10 min, and then the light was turned off for 10 min. This was repeated six times. During this process, temperature changes of the AuNIF were measured, as shown in Figure 5b. Results of the reversibility of the photothermal effect indicated that for each period of light irradiation, the temperature of the AuNIF increased to >55.0 °C after irradiation for 10 min. The photothermal effect of the AuNIF displayed a good response with on/off light irradiation. The SEM image also indicated that the nanoislands of the AuNIF revealed no significant change after on/off light irradiation, as shown in Figure 5c. The photothermal effect of the AuNIF also displayed a good response to the on/off light irradiation. Overall, the photothermal effect of the reusable AuNIF demonstrated its potential application for the photothermal killing of bacteria.

**Figure 6:**
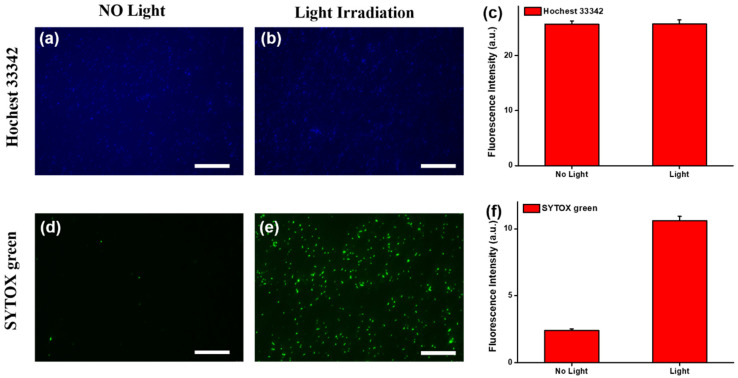
Fluorescence images of Hoechst 33342 for gold nanoisland films (AuNIFs) cultured with *Escherichia coli* (**a**) without or (**b**) with light irradiation. (**c**) Fluorescence intensities from (**a**,**b**). Fluorescence images of SYTOX green for AuNIFs cultured with *E. coli* (**d**) without or (**e**) with light irradiation. (**f**) Fluorescence intensities from (**d**,**e**). Scale bars are 50 μm.

To evaluate photothermal therapy, the AuNIF cultured with *E. coli* was irradiated with or without simulated AM1.5 sunlight. As shown in fluorescence images in Figure 6a,b, the blue fluorescence of Hoechst 33342 indicated that the total numbers of *E. coli* cultured on the AuNIF with or without light irradiation revealed no significant difference. The fluorescence intensities of Figure 6a,b were, respectively, calculated to be 25.71 and 25.76 based on measurements by ImageJ software (ImageJ 1.52a and accessed on 15.7.2021), as shown in Figure 6c. According to the fluorescence intensity of Hoechst 33342, the total number of *E. coli* cultured on the AuNIF with or without light irradiation were similar. Furthermore, the fluorescence of SYTOX green was applied to evaluate dead *E. coli*. As shown in Figure 6d,e, fluorescence images clearly showed that with light irradiation, the intensity of SYTOX green fluorescence was higher than that without light irradiation. The fluorescence intensities of Figure 6d,e were separately calculated to be 2.4 and 10.6, respectively, as shown in Figure 6f. The fluorescence intensity of the AuNIF cultured with *E. coli* with light irradiation was 4.4-fold higher compared to that without light irradiation. With light irradiation, the plasmonic AuNIF absorbed light and then transformed it into heat by a photothermal effect, resulting in the photothermal killing of *E. coli*. Results of the photothermal therapy demonstrate that the plasmonic AuNIF could be a promising substrate for antibacterial applications.

## 4. Conclusions

A facile seed-mediated growth approach was applied to deposit an AuNIF onto a glass substrate. The structure of the gold nanoislands, element distribution, and plasmonic absorption of the AuNIF were, respectively, demonstrated by SEM images, an EDX analysis, and UV-Vis spectrum. The capture efficiency of *E. coli* on the surface of the AuNIF was 16.7-fold greater compared to that of the bare glass substrate due to the surface topography of the nanoislands. For the SERS application, Raman signals of *E. coli* cultured on the surface of plasmonic AuNIF were greatly enhanced compared to those on the bare glass substrate. Regarding the photothermal effect, the temperature of the AuNIF increased to 60.0 °C after light irradiation at a power density of 300 mW/cm^2^ for 15 min. The AuNIF also exhibited excellent reversibility of the photothermal effect and a good response to on/off light irradiation. As for photothermal therapy, the AuNIF revealed a higher efficiency for the photothermal killing of *E. coli* compared to that of the glass substrate. This work demonstrated that plasmonic AuNIF could be a potential platform for bacterial theranostics.

## Figures and Tables

**Table 1:** Raman peaks of the surface-enhanced Raman scattering (SERS) spectrum of *Escherichia coli* on the gold nanoisland film (AuNIF).

Raman Peaks	Assignment
744	Adenine, glycosidic ring mode
911	υ(C-COO^−^) (carbohydrates)
1115	CC skeletal and υ(COC) from glycosidic link (carbohydrates)
1221	Amide III, adenine, polyadenine and DNA
1321	υ(NH_2_) adenine, polyadenine, DNA
1368	υ(COO^−^) and δ(C-H) proteins
1453	CH_2_ deformation mode of proteins.
1585	Tyrosine
1623	Amide I (unsaturated lipids)

The modes: υ, stretch; δ, bend.

## Data Availability

The data presented in this study are available on request from the corresponding author.
